# Growth Patterns of *Clostridium difficile* – Correlations with Strains, Binary Toxin and Disease Severity: A Prospective Cohort Study

**DOI:** 10.1371/journal.pone.0161711

**Published:** 2016-09-06

**Authors:** Sarah Tschudin-Sutter, Olivier Braissant, Stefan Erb, Anne Stranden, Gernot Bonkat, Reno Frei, Andreas F. Widmer

**Affiliations:** 1 Division of Infectious Diseases and Hospital Epidemiology, University Hospital Basel, Basel, Switzerland; 2 Laboratory of Biomechanics and Biocalorimetry, University of Basel, Basel, Switzerland; 3 Department of Urology, University Hospital Basel, Basel, Switzerland; 4 Division of Clinical Microbiology, University Hospital Basel, Basel, Switzerland; Cleveland Clinic, UNITED STATES

## Abstract

A broad spectrum of symptoms has been associated with *C*. *difficile* infection (CDI). Several studies indicate that toxin-production correlates with growth rates of *C*. *difficile*. This study aimed to correlate growth rates of *C*. *difficile* with disease severity and strain characteristics. From 01/2003 to 10/2011, strains from a prospective cohort of all inpatients with CDI at the University Hospital Basel, Switzerland were analyzed regarding binary toxin, presence of the tcdC deletion and ribotype. Isothermal microcalorimetry was performed to determine growth rates, quantified by the Gompertz function. Ordered logistic regression models were used to correlate disease severity with strain features and clinical characteristics. Among 199 patients, 31 (16%) were infected with binary toxin-producing strains, of which the tcdC gene-deletion nt117 was detected in 9 (4%). Disease severity was classified as mild in 130 patients (65.3%), as severe in 59 patients (29.7%) and as severe/complicated in 10 patients (5.0%). Growth rates were inversely associated with disease severity in univariable (OR 0.514, 95%CI 0.29–0.91, p = 0.023) and multivariable analyses (OR 0.51, 95%CI 0.26–0.97, p = 0.040). While none of the strain characteristics such as presence of the tcdC gene deletion or binary toxin predicted CDI severity, growth rates were inversely correlated with disease severity. Further investigations are needed to analyze growth-regulators and respective correlations with the level of toxin production in *C*. *difficile*, which may be important determinants of disease severity.

## Introduction

*Clostridium difficile* is the most frequent cause of healthcare associated diarrhea, and a significant cause of morbidity and mortality among hospitalized patients [1]. In a recently published multistate prevalence survey of health care–associated infections in the United States, *C*. *difficile* was the most commonly reported pathogen causing 12.1% of health care–associated infections–underscoring its ongoing significance in modern times [2]. A broad spectrum of clinical symptoms ranging from mild diarrhea to severe toxic colitis has been associated with *C*. *difficile* infection (CDI). Differences in disease presentation and severity have been attributed to both host- and strain-related factors. While age, comorbidities and prior exposure to antibiotics are important predictors of disease severity [3], hyperproduction of toxins A and B–the primary virulence factors of *C*. *difficile*–and production of binary toxin have been suggested as strain-related risk factors [3–5]. However, results regarding the independent contribution of strain-characteristics have been conflicting [6, 7] and questioned in non-epidemic settings [8–11].

Previous studies pointing to associations between toxin-hyperproduction (partially conferred by a deletion in the tcdC gene) and disease-severity have shown that the production of toxins A and B is co-regulated and growth-dependent [12]. While the logarithmic phase of growth kinetics is associated with weak transcription of genes encoding for toxin A and B, the inverse is observed in the stationary phase [12].

Isothermal microcalorimetry measures heat flow related to chemical or physical processes with high resolution [13]. By detection of less than a microwatt of heat flow, heat produced by microorganisms in relation to their metabolic activity and replication rates (ranging from 0.2-500uW) can be measured continuously and translated into a real-time electronic signal, reflecting growth-related heat flow patterns [13, 14].

We sought to correlate growth rates of *C*. *difficile* as determined by isothermal microcalorimetry with disease severity and strain characteristics, in particular presence of the tcdC deletion and the gene encoding for binary toxin.

## Methods

### Setting

The University Hospital Basel is a tertiary academic medical care center in Switzerland, with 855 beds, admitting more than 32,000 adult patients per year. The study was approved by the institutional review board (Ethikkommission Nordwest-und Zentralschweiz, EKNZ) as part of the quality assurance program, and informed consent was waved.

### Patients and data collection

In January 2003, an ongoing prospective cohort of all inpatients diagnosed with CDI was established and respective strains were cultured and saved as detailed below. For this study, two strains (and respective patients) were randomly selected each month from January 2003 to October 2011, using a computer-generated random number list.

The selection was performed by a member of the Division of Infectious Diseases and Hospital Epidemiology of the University Hospital Basel not involved in this study. Random selection was stratified by each month included in the study period.

A CDI episode was defined according to as a clinical picture compatible with CDI and microbiological proof of free toxins and the presence of *C*. *difficile* in stool without reasonable evidence of another cause of diarrhea; or pseudomembranous colitis as diagnosed during endoscopy (with or without confirmation by histopathological findings), after colectomy or on autopsy [15].

Clinical characteristics including disease severity (defined as mild, severe and severe/complicated) adapted from the ESCMID guidelines were collected [15]. Severe CDI was defined as a CDI episode with one or more specific signs and symptoms of severe colitis and complicated CDI was defined as a course of disease, with significant systemic toxin effects and shock, resulting in need for ICU admission, colectomy or death [15]. Patients without severe colitis or complicated course were considered having mild disease. Information on exposure to antibiotics, receipt of chemotherapy or immunosuppressants, and use of proton pump inhibitors was captured. Exposures were collected if they occurred ≤ 7 days prior to CDI onset, except for antibiotics, which were captured ≤ 30 days prior to CDI onset and chemotherapy, which was captured ≤ 3 months prior to CDI onset.

### *C*. *difficile* laboratory diagnostics

From 2003 to 2007, stool specimens submitted for *C*. *difficile* detection were analyzed by fecal toxin A/B by enzyme immunoassay (CDIFF TOX A/B II; TechLab/Wampole, Blacksburg, VA) directly from stool (toxin-EIA) and toxigenic culture (TC) when fecal-toxin was negative. In 2008, screening for *C*. *difficile* glutamate dehydrogenase antigen (Wampole® C.DIFF CHEKTM-60) was introduced and only positive stools were further evaluated for *C*. *difficile* toxin by EIA or TC [16].

From 2003, anaerobic cultures were performed for all stool samples tested positive for toxigenic *C*. *difficile* and all strains were prospectively collected and saved at -70°C. All specimens were plated on selective cycloserine-cefoxitin-blood agar plates (CLO agar; bioMérieux, Marcy l’Etoile, France) without any delay and incubated in an anaerobic chamber for 48 hours according to standard laboratory methods. *C*. *difficile* colonies were identified on the basis of their typical morphology on agar plates and by the gram strain, characteristic odour, and unique pattern of fatty acid metabolic products by gas liquid chromatography [16].

All strains were randomly selected for this study and analyzed regarding the presence of the gene encoding for binary toxin and the tcdC deletion nt 117 (tcdCΔ117) (as determined by GeneXpert^®^). Polymerase chain reaction (PCR) ribotyping was performed for identification of ribotypes 027, 078 and 126 by using high-resolution capillary gel-based electrophoresis [17] for all strains with detection of the binary toxin-gene or the tcdC deletion. In brief, PCR was performed using the original principals developed by the Anaerobe Reference Unit in Cardiff, as described elsewhere [18]. Capillary electrophoresis was conducted using the automated sequencer ABI-PRISM^**®**^ 3130 Genetic Analyzer and fragments were analyzed using Applied Biosystems GeneMapper^**®**^ (Applied Biosystems) Software and GelCompar II (Applied Maths, Sint-Martens-Latem, Belgium) software.

### Measurement of growth rates by microcalorimetry

*C*. *difficile* isolates were cultured anaerobically on pre-reduced fastidious anaerobic agar (FAA; Lab M Ltd, Bury, UK) at 37°C for 48 h. Colonies were subsequently suspended in Tryptone Soya Broth (TSB, Oxoid Ltd, Basingstoke, UK) to a turbidity of 4.0, using the McFarland scale. They were then adjusted using phosphate-buffered saline to an optical density at 600 nm and used to inoculate the samples at 0.01%.

To assess comparability of growth rates as determined by microcalorimetry on different media, 10 strains were suspended into another commercial growth media (Cycloserine Cefoxitin Mannitol Broth with Taurocholate and Lysozyme, CCMB-TAL, Anaerobe Systems, Morgan Hill CA, USA).

A microcalorimetry thermostat (Thermal Activity Monitor, Model 3102 TAMIII, TA Instruments, New Castle, DE) equipped with 48 channels was used to measure the heat flow (power-time) curves. In isothermal mode, the liquid in the thermostat was maintained at the set temperature 37.0°C with an absolute accuracy of 0.02°C. Any heat generated or absorbed by the sample was measured continuously over time. After the initial equilibration time of 15 minutes, heat flow rates were recorded over 5 days at 10 second intervals.

Three ml of liquid culture of *C*. *difficile* was introduced in 4ml microcalorimetric ampoules. Then the ampoules were sealed and the headspace flushed with nitrogen to remove any oxygen that might be present. To check the anaerobic conditions in the ampoules resazurin was added to selected samples. After flushing, samples were introduced in the microcalorimeter and measurements were performed. Total heat was determined by integration of the area between the heat flow-time curve and the baseline at two places. Data analysis was accomplished with the manufacturer’s software (TAM Assistant), R (R Development Core Team) and the grafit package. Maximum growth rate were estimated from the complete microcalorimeteric curve using the Gompertz model [19]. All microcalorimetric analyses were performed blinded from the clinical context.

Optical density (OD600nm) readings were used to validate growth rates determined by microcalorimetry.

### Statistical analyses

The primary outcome measures was disease severity classified as mild, severe and severe/complicated. The secondary outcome was the maximum growth rate quantified by the Gompertz model.

Patients were categorized into three groups based on disease severity (i.e. mild, severe and severe/complicated CDI), which was the principal outcome measure. Ordered logistic regression models were used to correlate disease severity with strain features and clinical characteristics. All variables found to be significant in univariable analyses, as well as age—a known confounder in this setting—were included in the final multivariable regression model. In addition, we introduced a variable categorizing time by study years into the regression models to account for secular trends during the study period. The Brant test was performed to confirm the proportional odds assumption.

Univariable linear regression models were performed to identify associations between strain-related characteristics and growth rates (secondary outcome). The Shapiro-Wilk test was performed to test for normality of both growth rates and residuals. Differences between growth rates using two different media for performance of microcalorimetry, were compared by the Wilcoxon signed-rank test. Two-sided p-values <0.05 were considered statistically significant. Analyses were performed using Stata statistical software, version 12.0 (Stata Corp, College Station, Texas).

## Results

From January 2003 to October 2011, 633 patients fulfilled the diagnosis of CDI. 212 cases were randomly selected by blocks of two cases of CDI per month. Strains were not available for four cases of CDI (two were missing in our strain collection and two could not be cultured after thawing) and clinical data was missing for two cases. Seven cases had to be excluded due to non-detectable growth of their respective strains as determined by microcalorimetry. These seven patients did not differ regarding baseline characteristics, prior exposures, strain characteristics (i.e. presence of the binary toxin-gene and tcdC deletion) or distribution of disease severity from the patients included in the analyses.

Among the remaining 199 patients, 130 (65.3%) were classified as having mild and 59 (29.7%) as having severe CDI. Severe complicated CDI was diagnosed in 10 cases (5.0%). Patients’ baseline characteristics and prior exposures are summarized in Table 1. In univariable analyses, none of the clinical characteristics was associated with disease severity in this cohort, except for gender (Table 1).

**Table 1:** Associations of clinical characteristics with disease severity.

Associations of clinical characteristics with disease severity						
	Mild CDI (n = 130)	Severe CDI (n = 59)	Severe/complicatedCDI (n = 10)	OR	95%CI	p-value
**Patient characteristics**						
Male	70 (53.8%)	20 (33.9%)	5 (50%)	0.52	0.28–0.93	**0.029**
Age	65.8 (+/-16.1)	66.4 (+/-16.4)	67.2 (+/-9.2)	1.00	0.98–1.02	0.739
Number of comorbidities	8 (0–10)	8 (1–10)	8 (5–8)	0.93	0.83–1.04	0.217
Charlson comorbidity index	2 (0–11)	2 (0–8)	2 (0–9)	0.94	0.82–1.07	0.320
Immunosuppression	16 (12.3%)	3 (5.1%)	0 (0.0%)	0.32	0.09–1.15	0.081
Bone marrow transplant	11 (8.5%)	0 (0.0%)	0 (0.0%)	N/A	N/A	N/A
Renal transplant	5 (3.8%)	2 (3.4%)	0 (0.0%)	0.71	0.14–3.66	0.680
Dialysis	5 (3.8%)	1 (1.7%)	1 (10.0%)	0.87	0.16–4.71	0.872
** Prior exposures**						
Antibiotics	122 (93.8%)	56 (94.9%)	9 (90%)	1.14	0.21–6.15	0.883
Steroids	11 (8.5%)	2 (3.4%)	0 (0.0%)	0.32	0.07–1.50	0.149
Chemotherapy	19 (14.6%)	7 (11.9%)	0 (0.0%)	0.64	0.26–1.60	0.342
Proton pump inhibitors	87 (66.9%)	31 (52.5%)	6 (66.7%)	0.60	0.32–1.12	0.106

CDI: *Clostridium difficile* infection

OR: Odds ratio

CI: Confidence interval

Significant p-values are printed in bold

Thirty-one patients were infected with binary toxin-producing strains. The deletion in the tcdC gene was identified in 9 strains. PCR ribotypes 027, 078 and 126 were detected in 5, 7, and 8 cases, respectively. All 5 strains identified as PCR ribotype 027 exhibited the tcdC gene deletion. While none of the strain-related characteristics were associated with disease severity, direct detection of toxin from stool was related to more severe CDI manifestations (Table 2).

**Table 2:** Associations of strain characteristics with disease severity.

Associations of strain characteristics with disease severity						
	Mild CDI (n = 130)	Severe CDI (n = 59)	Severe/complicated CDI (n = 10)	OR	95%CI	p-value
**Strain characteristics**						
Binary toxin	21 (16.2%)	9 (15.3%)	1 (10%)	0.86	0.38–1.93	0.715
tcdC deletion	5 (3.9%)	4 (6.8%)	0 (0.0%)	1.36	0.37–4.94	0.642
PCR ribotype 027	2 (1.5%)	3 (5.1%)	0 (0.0%)	2.25	0.46–11.34	0.326
PCR ribotype 078	7 (5.4%)	0 (0.0%)	0 (0.0%)	N/A	N/A	N/A
PCR ribotype 126	5 (3.9%)	3 (5.1%)	0 (0.0%)	1.040	0.25–4.32	0.956
**Direct detection of toxin from stool**	100 (76.9%)	51 (86.4%)	10 (100.0%)	2.38	1.03–5.50	**0.042**

CDI: *Clostridium difficile* infection

PCR: Polymerase chain reaction

OR: Odds ratio

CI: Confidence interval

Significant p-values are printed in bold

**Fig 1:**
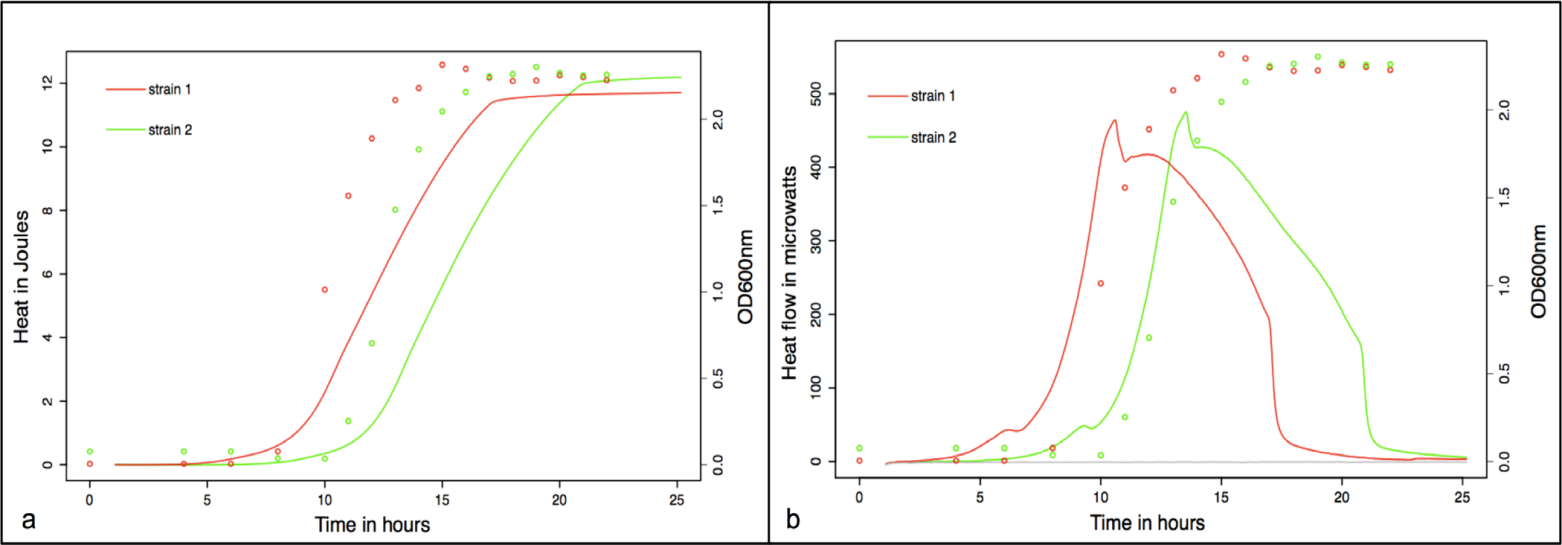
**a)** Correlation of heat in joules (as determined by microcalorimetry) and optical density (OD600nm) readings over time for two different strains of toxigenic *C*. *diffcile*. **b)** Correlation of heat flow in microwatts and OD600nm over time. Heat measurements are indicated by lines and OD600nm readings are indicated by dots) for two different strains of toxigenic *C*. *diffcile* (indicated by red and green).

Growth rates as determined by heat measurements (in joules, indicated by lines in Fig 1) for two different strains of toxigenic *C*. *difficile (distinguished by the colors red and green)* were compared to OD600nm readings over time (indicated by dots) and showed good correlation (Fig 1A). Comparisons of heat flow (measured in microwatts and indicated by lines) to OD600nm readings (indicated by dots), reveal that a substantial part of the second heat flow peak is not related to growth (Fig 1B). Growth rates were normally distributed (p = 0.117), had a median of 1.17 (IQR 0.83–1.50), a mean of 1.18 (standard deviation +/- 0.53) and did not differ depending on the growth media used (p = 0.680).

In univariable linear regression analyses, none of the strain-related characteristics as presence of tcdC gene deletion, binary toxin or PCR ribotypes were associated with growth rates quantified by the Gompertz function. Furthermore, no association between direct detection of toxins in stool and growth rate was identified (Table 3). All residuals of the different univariable regression models were normally distributed indicating adequate model fit.

**Table 3:** Analyses of growth rates according to different strain characteristics.

Analyses of growth rates according to different strain characteristics			
	Effect estimate	95%CI	p-value
**Strain characteristics**			
Binary toxin	-0.11	-0.31–0.10	0.313
tcdC deletion	-0.08	-0.43–0.28	0.672
PCR ribotype 027	0.05	-0.43–0.52	0.852
PCR ribotype 078	-0.12	-0.52–0.28	0.567
PCR ribotype 126	-0.30	-0.68–0.07	0.112
**Direct detection of toxin from stool**	-0.01	-0.20–0.17	0.890

Differences in mean growth rates were determined by univariable linear regression

PCR: Polymerase chain reaction

CI: Confidence interval

**Fig 2:**
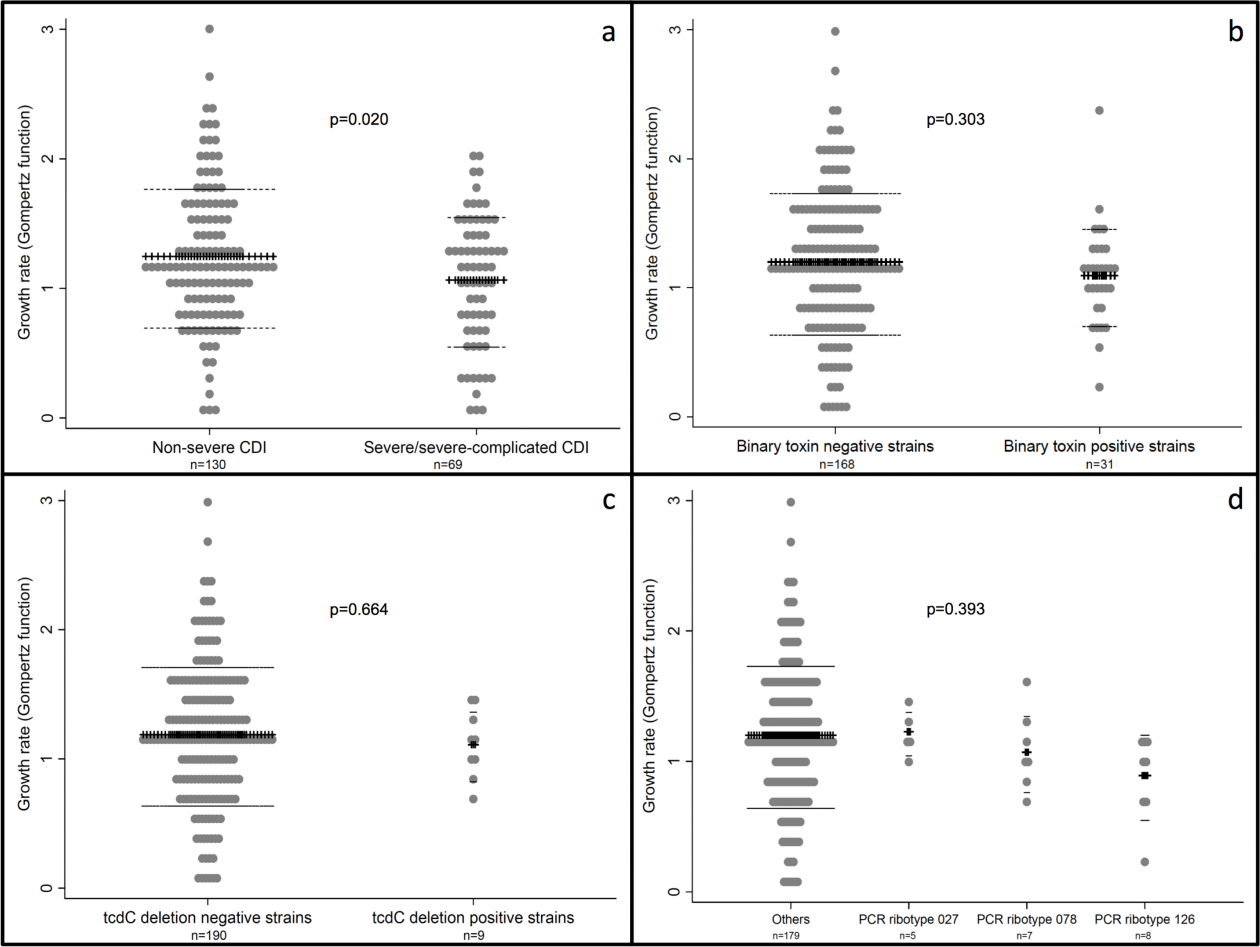
Growth rates of *C*. *difficile* as determined by microcalorimetry and quantified by the Gompertz function according to disease severity (a), presence of the gene encoding for binary toxin (b), presence of tcdC gene deletion (c) and different strain types (d).

Growth rates quantified by the Gompertz function were inversely associated with disease severity in both univariable (OR 0.514, 95%CI 0.29–0.91, p = 0.023) and multivariable analyses (OR 0.51, 95%CI 0.26–0.97, p = 0.040), adjusting for possible confounders (age, male gender and toxin detection from stool) (Table 4). The Brant test revealed insignificant p-values (0.363–0.961) confirming the proportional odds assumption. Fig 2 summarizes growth rates of *C*. *difficile* according to disease severity (a), presence the gene encoding for binary toxin (b), presence of tcdC gene deletion (c) and different strain types (d).

**Table 4:** Crude and adjusted odds ratios for CDI severity.

Crude and adjusted odds ratios for CDI severity						
	Crude			Adjusted		
	OR	95% CI	p-value	OR	95% CI	p-value
Growth rate	0.51	0.29–0.91	**0.023**	0.51	0.26–0.97	**0.040**
Age	1.00	0.98–1.02	0.739	1.01	0.99–1.03	0.521
Male gender	0.52	0.28–0.93	**0.029**	0.42	0.22–0.80	**0.008**
Direct detection of toxin from stool	2.38	1.03–5.50	**0.042**	2.70	1.13–6.44	**0.025**
Time (categorized by study year)	0.86	0.77–0.97	**0.013**	0.88	0.77–0.99	**0.039**

CDI: *Clostridium difficile* infection

OR: Odds ratio

CI: Confidence interval

Significant p-values are printed in bold

## Discussion

Growth rates were inversely correlated with disease severity, while no other strain characteristics as presence of the tcdC gene deletion, binary toxin or ribotype predicted severe CDI in this cohort. Further, growth rates were not related to specific strain determinants. To our knowledge, no other studies have related growth rates of *C*. *difficile* as determined by microcalorimetry with CDI severity and different strain characteristics so far. Our results may seem conflicting as disease severity may be thought to be associated with rapidly expanding strains resulting in increased bacterial load and concomitant toxin production. However, our findings are supported by a recently published study examining 106 *C*. *difficile* isolates, describing slower growth rates (measured by optical density readings) for ribotype 027 and reporting that isolates from severe CDI cases did not appear to grow more rapidly than others [20]. Furthermore, the authors described a negative correlation between growth rates and toxin production, with isolates producing higher levels of toxin also growing slower–a finding that remained significant after excluding 027 isolates from their analysis indicating its independence of specific ribotypes [20]. The authors hypothesized that possibly a shift in overall metabolism allows isolates to better commit resources to the production of toxin [20] a theory supported by the fact that toxin-production has been shown to occur in the stationary phase of *C*. *difficile* growth kinetics [12]. In accordance with our results, they concluded that hypervirulence might not be related to specific strain types. While toxin production was not measured in our study, direct toxin detection from stool rather than culture-suggesting higher toxin concentration- was associated with CDI severity in our study–a result supported by a recently published study describing the correlation of cytotoxin assay positivity with clinical outcome [21]. Comparisons of heat flow to optical density readings in our study, indicate that a substantial part of heat flow is not related to growth, suggesting alternate metabolic activity–possibly toxin production.

It is noteworthy, that growth rate–a strain related feature- was correlated with disease severity in our study, despite the low numbers of hypervirulent strains detected in this cohort. Such findings suggest that other strain-characteristics may be of greater importance in conferring virulence than those commonly investigated and reported to date. Established determinants of hypervirulence as binary toxin and tcdC gene deletion were not associated with disease severity in our cohort. Findings, which are consistent with those from other studies, reporting on lacking associations between tcdC and more severe disease [22, 23]. A correlation between binary toxin and severity of diarrhea has been suggested, but has been questioned [24].

No associations of the hypervirulent ribotypes 027 and 078 with more complicated CDI were observed in a large European survey including 389 patients with CDI and characterization of their respective strain-type [25]. This finding is supported by a US-study also challenging the concept of disease severity being linked to hypervirulent strains as the association between ribotypes 027 and 078 and severe CDI was not significant after correcting for any of the other clinical covariates [6].

Limitations of our study are (i) Its performance at a single center and the low incidence of hypervirulent strains limit the generalizability to other settings. (ii) our study is underpowered to draw conclusions regarding associations with disease severity and growth rates for specific ribotypes due to the small numbers of *C*. *difficile* strains belonging to PCR ribotypes 027 and 078 at our institution. Standardized test to evaluate the level of toxin production are lacking, and therefore, we did not perform quantitative determinations of toxin production. Correlating toxin production with disease severity, strain characteristics and growth rates require further investigation. (iv) Exposures to known risk factors for development of CDI were only collected during defined time-frames as outlined in the methods section to avoid recall-bias, therefore exposure to risk factors known to increase the risk for CDI beyond the chosen time-frame is not accounted for in this study.

In conclusion, slower rate of growth was significantly associated with CDI severity Growth rates were not related to specific determinants of hypervirulence as presence of the tcdC gene deletion or binary toxin. Further investigations are needed to analyze growth-regulators and their correlation with toxin production in *C*. *difficile*, which both may be more important determinants of disease severity.

## Supporting Information

S1 File (XLSX)
